# Advances in Cyclosporiasis Diagnosis and Therapeutic Intervention

**DOI:** 10.3389/fcimb.2020.00043

**Published:** 2020-02-11

**Authors:** Junqiang Li, Zhaohui Cui, Meng Qi, Longxian Zhang

**Affiliations:** ^1^Academy of Chinese Medical Sciences, Henan University of Chinese Medicine, Zhengzhou, China; ^2^College of Animal Science and Veterinary Medicine, Henan Agricultural University, Zhengzhou, China; ^3^College of Animal Science, Tarim University, Alar, China

**Keywords:** cyclosporiasis, clinical features, detection methods, prevention, therapy

## Abstract

Cyclosporiasis is caused by the coccidian parasite *Cyclospora cayetanensis* and is associated with large and complex food-borne outbreaks worldwide. Associated symptoms include severe watery diarrhea, particularly in infants, and immune dysfunction. With the globalization of human food supply, the occurrence of cyclosporiasis has been increasing in both food growing and importing countries. As well as being a burden on the health of individual humans, cyclosporiasis is a global public health concern. Currently, no vaccine is available but early detection and treatment could result in a favorable clinical outcome. Clinical diagnosis is based on cardinal clinical symptoms and conventional laboratory methods, which usually involve microscopic examination of wet smears, staining tests, fluorescence microscopy, serological testing, or DNA testing for oocysts in the stool. Detection in the vehicle of infection, which can be fresh produce, water, or soil is helpful for case-linkage and source-tracking during cyclosporiasis outbreaks. Treatment with trimethoprim-sulfamethoxazole (TMP-SMX) can evidently cure *C. cayetanensis* infection. However, TMP-SMX is not suitable for patients having sulfonamide intolerance. In such case ciprofloxacin, although less effective than TMP-SMX, is a good option. Another drug of choice is nitazoxanide that can be used in the cases of sulfonamide intolerance and ciprofloxacin resistance. More epidemiological research investigating cyclosporiasis in humans should be conducted worldwide, to achieve a better understanding of its characteristics in this regard. It is also necessary to establish *in vitro* and/or *in vivo* protocols for cultivating *C. cayetanensis*, to facilitate the development of rapid, convenient, precise, and economical detection methods for diagnosis, as well as more effective tracing methods. This review focuses on the advances in clinical features, diagnosis, and therapeutic intervention of cyclosporiasis.

## Introduction

Cyclosporiasis is caused by *Cyclospora cayetanensis*, and in humans it typically induces periodic profuse watery diarrhea (Shields and Olson, 2003a; Ortega and Sanchez, 2010; Almeria et al., 2019; Giangaspero and Gasser, 2019). Human *C. cayetanensis* infection has been documented in over 56 countries worldwide, and 13 of these have recorded cyclosporiasis outbreaks (Li et al., 2019a). The latest large scale cyclosporiasis outbreaks occurred in 2013 and 2018 in multiple states of the US (Abanyie et al., 2015; Casillas et al., 2018).

As of today, 22 *Cyclospora* species are identified in humans and various animals, including vipers, moles, myriapodes, rodents, and monkeys (Li et al., 2015a, 2019a; McAllister et al., 2018). *C. cayetanensis* is the only documented *Cyclospora* species known to infect humans (Ortega and Sanchez, 2010; Li et al., 2019a). The overall prevalence of *C. cayetanensis* was 3.6% in humans worldwide (Li et al., 2019a). Of the other species, *C. papionis* was detected in 17.9% of the captured baboons in Kenya (Li et al., 2011) and *C. macacae* in 6.8% of the rhesus monkeys in China (Li et al., 2015b). In humans, most of these infections are contracted via the fecal-oral route, and water, berries, basil, cilantro, and other food produce can be a vehicle for *Cyclospora* transmission (Almeria et al., 2019). The *Cyclospora* infection is evidenced to be linked with consumption of contaminated food and water or contact with transmission vehicles of oocysts (Li et al., 2019a).

Although large outbreaks of cyclosporiasis have been documented in developed countries, *C. cayetanensis* infections are most commonly reported in developing countries or in endemic areas (Li et al., 2019a). In susceptible individuals, cyclosporiasis is reported to be most prevalent in immunocompetent diarrheic patients (Li et al., 2019a). There are notable seasonal distributions of *C. cayetanensis* infections that commonly occur in rainy or summer season (Zhou et al., 2011; Kaminsky et al., 2016). Cyclosporiasis causes significant health problem to the people traveling or expatriating to the under developed or developing countries having poor sanitation and high population density (Fryauff et al., 1999; Mansfield and Gajadhar, 2004; Pandey et al., 2011; Kłudkowska et al., 2017).

The entire genome of *C. cayetanensis* had been sequenced (Liu et al., 2016; Qvarnstrom et al., 2018), and there have been recent improvements in detection methods and therapeutic interventions for cyclosporiasis. This review presents an update on aspects of the clinical features, detection methods, therapy, and prevention of cyclosporiasis.

## Clinical Features

### Intestinal Infection Features

#### Clinical Symptoms

Frequently the cyclosporiasis patient is an immunocompetent traveler living in an industrialized country, returning from a tropical and/or developing country such as the Dominican Republic, Mexico, Guatemala, Haiti, Peru, or Nepal, among others. Typical symptoms on presentation include watery diarrhea, abdominal cramps, vomiting, anorexia, weight loss, and severe fatigue. Less frequently patients also report flu-like symptoms (Marques et al., 2017; Giangaspero and Gasser, 2019). Cyclosporiasis patients with immune dysfunction can experience severe, protracted, or chronic watery diarrhea along with nausea, abdominal pain, mild fever, lethargy, and emaciation (Field, 2002; Shields and Olson, 2003a; Mansfield and Gajadhar, 2004). The condition can be particularly challenging in organ transplant recipients undergoing immunosuppressive treatment (Giangaspero and Gasser, 2019).

Human cyclosporiasis can be asymptomatic, or range from mild to severe in endemic countries, such as Guatemala, Haiti, Peru, and Nepal (Mansfield and Gajadhar, 2004; Giangaspero and Gasser, 2019). The clinical outcomes of cyclosporiasis are related to the age and immune status of the host, endemicity in a specific area and some other unknown factors (Almeria et al., 2019). Infants and the elderly tend to exhibit more severe clinical symptoms, whereas milder or asymptomatic infections typically occur in older children and non-elderly adults (Giangaspero and Gasser, 2019).

The incubation period of *C. cayetanensis* infection ranges from 2 to 11 days, and the median incubation period is ~7 days (Almeria et al., 2019). The clinical symptoms usually resolve with the treatment by specific drugs. However, persistent infection is seen in untreated patients that can last for a few days to a month or even longer (Thapa and Basnyat, 2017). The mean duration of diarrhea caused by cyclosporiasis is longer in AIDS patients (199 days) compared to other patients (57 days) (Schubach et al., 1997; Sancak et al., 2006; Ortega and Sanchez, 2010).

#### Endoscopic Change

In a previous study involving endoscopy of 17 Peruvian cyclosporiasis patients, moderate to marked erythema was observed in the distal duodenum of some cases and mild to moderate inflammation in the intestinal lamina propria of the other cases (Ortega et al., 1997). However, ulcer or hemorrhage like gross abnormalities were absent in both stomach and small intestine in any of the cyclosporiasis patients (Ortega et al., 1997).

#### Histology Change

Striking intestinal histological changes have been observed in patients with cyclosporiasis. Alteration of the overall architecture of the intestinal mucosa has been reported, with dramatic shortening of the intestinal villi and disruption of the surface epithelium (Ortega et al., 1997; Ortega and Sanchez, 2010). Villous atrophy and crypt hyperplasia in the duodenum and ileum have also been described (Connor et al., 1993; Field, 2002). In the aforementioned 17 Peruvian patients there was reactive hyperemia with vascular dilatation and congestion of villous capillaries (Ortega et al., 1997), and some patients have exhibited variably increased chronic inflammatory cells and intense lymphocytic infiltration in the lamina propria and epithelial tissue (Ortega et al., 1997; Wiwanitkit, 2006). Ortega et al. (1997) also reported extensive infiltration of lymphocytes into the surface epithelium, which was particularly prominent at the tip of the shortened villi. In another report, the *C. cayetanensis* induced inflammatory reactions were found to be lasted even after clearance of parasitic infection (Connor et al., 1999). Notably however, the pathogenesis underlying these symptoms has not been defined.

#### Intracellular Changes

Increases in lymphocytes in the intestinal surface epithelium have been reported during oocyst infections, during which parasitophorous vacuoles containing *C. cayetanensis* at various stages of the sexual and asexual life-cycle were observed in the apical cytoplasm of the enterocytes overlying the tips of the villi (Field, 2002). Via electron microscopy, rounded or more mature elongated fusiform merozoites up to 6 μm in length can be seen stacked in vacuoles within enterocytes, as well as occasional micro-gametocytes and macro-gametocytes (Field, 2002).

In high-magnification light microscopic examination, the parasite was seen at the luminal surface and the glandular clefts (Ortega et al., 1997) with having two completely developed asexual forms, Type I and II meronts. About 8–12 fully mature merozoites (~0.5 × 3–4 μm) were observed in Type I meront while 4 fully differentiated merozoites (~0.7–0.8 × 12–15 μm) were in Type II meront (Ortega et al., 1997). The parasite was also observed to have sexual forms, such as gametocytes. As typically seen in other coccidia, the merozoites of both Type I and II meronts contained rhoptries, micronemes, and nuclei. Meanwhile, the characteristic wall-forming body types I and II and polysaccharide granules were observed in macro-gametocytes of the parasite (Ortega et al., 1997). However, the gastric antral biopsy could not detect the *Cyclospora* parasite (Ortega et al., 1997). The findings of light microscopic examination were all substantiated by the transmission electron microscopy of the both asexual and sexual forms of the parasite.

### External Features of Infection

*Cyclospora cayetanensis* oocysts were evidenced to infect extraintestinal tissue such as biliary tract (Sifuentes-Osornio et al., 1995), resulting in acalculous cholecystitis in an AIDS patient (Zar et al., 2001). The pathogenesis of biliary infections is unknown. Presumably sporozoites from the intestinal lumen travel to bile ducts and initiate the development of *Cyclospora* there (Almeria et al., 2019).

Although *C. cayetanensis* infection was not reported in the respiratory tract, oocysts were detected in the nasal secretion of two patients suffering from tuberculosis (Di Gliullo et al., 2000; Hussein et al., 2005). Additionally, *C. cayetanensis* infections were found to be linked with some other diseases, including Reiter syndrome (reactive arthritis syndrome) (Connor et al., 2001) and Guillain-Barre syndrome (Richardson et al., 1998).

## Detection in Stool

### Oocyst Morphology Detection

#### Wet Smears

Wet smears and conventional microscopy methods have been widely used to detect *C. cayetanensis* oocysts in clinical stool samples (Ortega and Sanchez, 2010). The smears can be made directly from fresh stools, or concentrated samples obtained from formalin-ether sedimentation or sucrose flotation techniques that enhance detection efficiency of small amount of *Cyclospora* oocysts in stool samples (Becker et al., 2013). When observed, *Cyclospora* oocysts in stool are easily identified as spherical and refractile entities that are 8–10 μm in size and have a central morula (Li et al., 2019a). Since the *Cyclospora* oocysts shed discontinously, manifold fecal samples (each at 2–3 days interval) should be collected in a week or more from a patient for accurate detection of oocysts (Yang et al., 2014). Wet smear examination is a simple, direct, rapid, and reliable method for visual detection of parasites, but it is costly and laborious, and it requires specific expertise. Given the size similarity to some other microorganisms such as *Cryptosporidium* (4–6 μm), *C. cayetanensis* should be further differentiated by staining on wet or dry mounts (Almeria et al., 2019).

#### Staining Tests

The oocyst walls of *Cyclospora, Cryptosporidium*, and *Cystoisospora* parasites have acid-fast lipids that help their detection by acid-fast staining (Garcia et al., 2017). Although the modified acid-fast stain test can be useful for identifying *Cyclospora* oocysts, variable levels of dye uptake may result in ghost oocysts, or pink-stained or poorly stained oocysts, or oocysts that are not stained at all and appear as non-refractile glassy spheres against the blue-green background, along with well-stained oocysts (deep red with a mottled appearance) (Ortega and Sanchez, 2010; Garcia et al., 2017; Almeria et al., 2019).

Modified acid-fast staining with further minor modifications are developed to improve *Cyclospora* detection, one of which is the use of 1% H_2_SO_4_ as a decolorizer (Garcia et al., 2017). Another modification is devised with addition of dimethyl sulfoxide to the phenol-basic fuchsine, and the inclusion of acetic acid with malachite green as a combined decolorizer counter-stain to achieve better penetration and thus enhanced visualization of the internal structures of oocysts (Garcia et al., 2017). Apart from this, other modified acid-fast staining methods, such as Ziehl-Neelsen acid-fast stain (Brennan et al., 1996; Clarke and McIntyre, 1996), modified Kinyoun's acid-fast stain (Gonçalves et al., 2005; Behera et al., 2008; Dillingham et al., 2009; Bhandari et al., 2015), and modified Kinyoun's carbolfuchsin stain (Alakpa et al., 2002; Chacín-Bonilla et al., 2007) are commonly used. Some other staining methods, including modified safranin stain (Visvesvara et al., 1997), trichrome stain (Turgay et al., 2007), and lacto-phenol cotton blue stain (Parija et al., 2003) were also found to be highly sensitive to detect *Cyclospora* oocysts in fecal smears.

Even with the aid of conventional staining methods for *Cyclospora* oocysts microscopic detection can be challenging (McHardy et al., 2014), but it remains the recommended diagnostic method. Of the various available stains, the modified Ziehl-Neelsen stain technique has been recommended for detecting *Cyclospora* oocysts in clinical samples (Brennan et al., 1996; Khanna et al., 2014).

#### Fluorescence Tests

*Cyclospora* oocysts' strong autofluorescence properties render fluorescence microscopy useful for identification (Garcia et al., 2017). On 365- and 450–490-nm ultraviolet light exposures, the oocysts appear blue and green, respectively (Ortega and Sanchez, 2010; McHardy et al., 2014). In epifluorescence microscopic examination using a 330–380-nm ultraviolet filter, *C. cayetanensis* oocysts were observed to be easily visible in clinical samples (Eberhard et al., 1997), which enhances detection at least 2-fold over direct wet mounts, particularly in cases where the mounts or stained slides contain few oocysts (Berlin et al., 1998). The autofluorescence technique is reportedly markedly superior to wet smears and staining procedures for *Cyclospora* oocyst detection (Berlin et al., 1998).

#### Flow Cytometry

On the basis of morphological and autofluorescence properties of oocysts, a flow cytometry detection assay was developed for *C. cayetanensis* (Dixon et al., 2005). While the sample preparation time for flow cytometry is slightly longer than that for microscopy, the actual analysis time is much shorter. Furthermore, the flow cytometry is mostly automated that omits the problems of technical experience and tiresomeness of other manual analyses which may affect the results of detection (Li et al., 2014). A comparative study for the detection and quantification of *Cyclospora* oocysts observed no significant differences between flow cytometry and quantitative real-time PCR assays (Hussein et al., 2007).

To sum up, among the various available detection methods of *Cyclospora* oocyst based on morphology, the modified Ziehl-Neelsen staining and autofluorescence techniques have been recommended for the detection of oocysts in the clinical samples (Berlin et al., 1998; Khanna et al., 2014).

### Serological Tests

Serological screening for *Cyclospora* would facilitate epidemiological studies, especially in outbreak investigations (Ortega and Sanchez, 2010), but currently commercial serological assays to identify human exposure to *Cyclospora* are not available (Almeria et al., 2019). There have been attempts to characterize human serological immune responses to cyclosporiasis, involving specific IgG and IgM antibodies being tested via enzyme-linked immunosorbent assays (Wang et al., 2002), but specific diagnosis of infection at the individual patient level has not been achieved (Giangaspero and Gasser, 2019). The main constrain is the unavailability of suitable *Cyclospora* culture method that can be used for the propagation of oocysts and also the in-depth study of various aspects of the parasite (Eberhard et al., 2000; Cinar et al., 2015).

### Molecular Detection

Over the few decades, several conventional, nested, and quantitative PCR (qPCR) as well as multiplex PCR assays (together with other parasites) are developed for the identification of *Cyclospora* (Relman et al., 1996; Varma et al., 2003; Taniuchi et al., 2011; Li et al., 2015a, 2019a; Almeria et al., 2019) (Table 1). There is one commercially available fully automated system involving high-order multiplex PCR reactions that is capable of detecting *C. cayetanensis* with high sensitivity and specificity (Buss et al., 2015). The multiplex Biofire (Salt Lake City, UT, USA) FilmArray Gastrointestinal Panel is a commercially available DNA-based technology for the detection of *C. cayetanensis* (Ryan et al., 2017; Hitchcock et al., 2019).

**Table 1:** Common *Cyclospora cayetanensis* detection methods.

**Methods**	**Characteristics**	**LoD (limit of detection)**	**References**
Wet smears using light microscopy	Smears from fresh or concentrated feces; oocysts identified as 8- to 10-μm, spherical, refractile, with a central morula, and resembling wrinkled cellophane	Low, usually detection with other methods	Zhou et al., 2011; Becker et al., 2013
Modified acid-fast stain	A series of modified acid-fast staining methods, such as: ziehl-neelsen, Kinyoun's, carbolfuchsin, etc. Some oocysts stain in deep red, whereas others stain pink, or remain unstained against the blue-green background	Recommended for diagnosis of clinical samples	Brennan et al., 1996; Alakpa et al., 2002; Gonçalves et al., 2005; Dillingham et al., 2009
Modified safranin stain	Oocysts reddish orange stained, and the cyst wall more clearly with background	It is reported being superior to acid-fast stain, with fast, reliable, and easy to perform	Visvesvara et al., 1997
Lacto-phenol cotton blue (LPCB) stain	Oocysts stained in blue, and internal structures is clear	Recommended, if the acid-fast stain is not performed	Parija et al., 2003
Fluorescence microscopy	Oocysts autofluorescence; they appear blue when exposed to 365 nm UV light and looks green under 450–490 nm excitation	At least 2-fold over the direct wet mount	Ortega and Sterling, 1996; Berlin et al., 1998; McHardy et al., 2014
Flow cytometry	Oocysts morphology and auto fluorescence features, with higher automation	No differences with qPCR assay for oocyst detection and counts	Dixon et al., 2005; Li et al., 2014
Serological test- ELISA	No commercial serological assays are available	Specific IgG and IgM antibodies needed for oocysts	Wang et al., 2002
Nested PCR molecular detection	Specific PCR primers for small subunit rRNA or ITS regions	One to 10 oocysts	Relman et al., 1996; Olivier et al., 2001; Li et al., 2007^*^
PCR-RFLP molecular detection	Use of restriction enzyme *Alu*I	As few as one oocyst in 10 liters water	Shields and Olson, 2003b
Quantitative PCR	Specific primers and probe	Estimate the DNA of 0.5 oocysts	Verweij et al., 2003
Quantitative PCR	Using the inherent genetic uniqueness of the 18S ribosomal gene sequence	As few as one oocyst per 5 μL reaction volume	Varma et al., 2003
Multiplex PCR	Simultaneous detection of *Cyclospora cayetanensis, Cystoisospora belli, Enterocytozoon bieneusi*, and *Encephalitozoon intestinalis*; detection of the amplicon is through specific probes coupled to Luminex beads	10^3^ plasmid copies	Taniuchi et al., 2011
Multiplex PCR	Commercially available DNA-based technologies for stool specimens	Simultaneous detection of 22 different enteric pathogens	Buss et al., 2015; Hitchcock et al., 2019
PCR assays and qPCR	Polymorphic junction region in the mitochondrial genome for human stool samples	As few as one oocyst	Guo et al., 2019; Nascimento et al., 2019
Multiplex qPCR and T4 phage internal control	Simultaneous detection of *Cryptosporidium parvum, Giardia lamblia*, and *Cyclospora cayetanensis* in human stool samples	20 copies: equivalently: 10^3^ of oocysts	Shin et al., 2018
FDA validated qPCR technique	FDA validated technique used in fresh produce matrices and prepared dishes	As few as five oocysts	Murphy et al., 2017, 2018b; Almeria et al., 2018
Multiplex qPCR	Highly specific, precise, and robust method that has potential for application in food-testing on berries	~10 oocysts	Temesgen et al., 2019a
PCR assay targeting the ITS	Potential for standard use in food testing, particularly berry fruits	~6.4 pg: equivalent to DNA of one oocyst	Temesgen et al., 2019b
Multiplex PCR	Simultaneous detection of protozoan (oo)cysts (*Cryptosporidium, Giardia, Cyclospora cayetanensis*, and *Toxoplasma gondii*) in leafy greens	1–10 oocysts/g spinach in 10 g samples processed	Shapiro et al., 2019

* *It was initially developed to analyze cattle samples, now it is widely used to analyze human samples*.

Recently, a molecular diagnostic method employing multiplex real-time PCR and a T4 phage internal control has been devised for the simultaneous detection of *Cryptosporidium parvum, Giardia lamblia*, and *C. cayetanensis* in human stools (Shin et al., 2018). The QIAstat gastrointestinal panel can detect a large range of acute gastroenteritis pathogens with a high sensitivity, including *C. cayetanensis* (Hannet et al., 2019). Molecular-based detection methods have the capacity to screen a number of organisms at a time with using multiplex platforms, and to detect them rapidly with high sensitivity (even detection of a single oocyst), thus overcoming some of the limitations of microscopy-based diagnosis. Now-a-days, the molecular techniques have been widely used in laboratory testing or verification of suspected clinical samples.

### Case-Linking and Tracking

Numerous genotyping methods for *C. cayetanensis* have been developed, and successfully used for epidemiological trace-back investigations of cyclosporiasis. Recently, a multilocus sequence typing (MLST) tool involving five microsatellite loci has been established and used for epidemiological source tracking of *C. cayetanensis* (Guo et al., 2016). There have many other potential uses of this MLST tool (Hofstetter et al., 2019). It has been used to investigate the population genetics of *C. cayetanensis* (Li et al., 2017; Guo et al., 2018). More recently, qPCR (Guo et al., 2019) and standard PCR assays (Nascimento et al., 2019) have been evolved for the genotyping of *C. cayetanensis* that use the polymorphic region of parasitic mitochondrial genome. Relationships have been corroborated by a significant number of epidemiological linkages, suggesting the usefulness of the technique for aiding epidemiological trace-back, case-linkage, source-tracking, and distinct case cluster investigations (Barratt et al., 2019; Guo et al., 2019; Nascimento et al., 2019), especially during cyclosporiasis outbreaks.

## Detection in Vehicles

### Fresh Produces

Usually only low numbers of protozoan oocysts exist in naturally contaminated produce. A very important step in the isolation process is the productive gaining of oocysts after a careful washing of the fresh produce (Shields et al., 2012; Li et al., 2019b). Different washing solutions have been used for the recovery of *C. cayetanensis* oocysts (Shields et al., 2012; Chandra et al., 2014; Lalonde and Gajadhar, 2016; Li et al., 2019b). A filter bag (BagPage®, Interscience Lab. Inc., Boston, MA) along with a commercial laboratory detergent (Alconox®, White Plains, NY) (Shields et al., 2012) is used as a valid wash protocol for the successful recovery of *C. cayetanensis* oocysts from fresh produce and thereby DNA extraction and a specific qPCR are followed for the detection of the parasite (Murphy et al., 2018a).

In recent years, several molecular techniques have been developed for the detection of *C. cayetanensis* in fresh produce, including qPCR and various multiplex qPCR methods (Steele et al., 2003; Lalonde and Gajadhar, 2011, 2016; Murphy et al., 2017; Shapiro et al., 2019) (Table 1). qPCR can steadily identify a few oocysts in the fresh produce, such as three oocysts in a gram of fruit, or five oocysts in a gram of herbs or green onions (Lalonde and Gajadhar, 2016). In other reports as few as five oocysts were detected in samples of raspberries, cilantro, parsley, basil, and carrots via a qPCR technique (Murphy et al., 2017, 2018b; Almeria et al., 2018). Other methods for *C. cayetanensis* detection in fresh produce have been developed, including a new multiplex qPCR technique that is highly specific, precise, and robust and has potential for application in food-testing laboratories (Temesgen et al., 2019a). The limit of detection of that technique was estimated to be 10 oocysts for *Cyclospora* organisms. Another qPCR assay targeting the internal transcribed spacer 1 region was developed for the detection of *C. cayetanensis* in berries, and proved to be an effective approach that may be a suitable option for use in food-testing laboratories (Temesgen et al., 2019b). A multiplex PCR assay has been developed for simultaneous detection of four protozoan oocysts via a rapid, inexpensive, and simple protocol (Shapiro et al., 2019).

Robertson et al. (2000) validated the use of lectin-coated paramagnetic beads for the isolation of *Cyclospora* oocysts from fruits and vegetables. As reported for the detection of *Cryptosporidium parvum*, specific antibody-coated beads can be used to isolate and concentrate the *C. cayetanensis* oocysts, but antibodies are not yet commercially available (Almeria et al., 2019).

### Water or Soil

There are many documented reports of *Cyclospora* oocysts contaminating water and soil derived from multiple countries (Sturbaum et al., 1998; Sherchand and Cross, 2001; Tram et al., 2008; Giangaspero et al., 2015; Bilung et al., 2017), and various techniques have been developed for the isolation and identification of *Cyclospora* oocysts from the environmental samples, such as water and soil (Quintero-Betancourt et al., 2002; Steele et al., 2003; Murphy et al., 2018b). As *Cryptosporidium* oocysts detection is performed by filtration or purification via immunomagnetic separation, followed by the labeling of oocysts with a specific fluorochrome and differential interference microscopy detection (Giangaspero and Gasser, 2019), *Cyclospora* oocysts can also potentially be detected. However, due to the lack of a specific antibody for *C. cayetanensis* oocysts, the probability can not be tested.

### Viability and Infectivity Tests

Determination of parasite viability and infectivity is important in clinical settings. Since there is no accurate assay for the evaluation of viability or infectivity of *C. cayetanensis*, it can be assessed via analysis of the sporulation rates of oocysts. It has been reported that *C. cayetanensis* oocysts complete sporulated in 2.5% potassium dichromate within 7–13 days at 25 or 32°C (Ortega et al., 1993, 1998). Unsporulated oocysts carry developing sporocysts, while sporulated oocysts carry two ovoid sporocysts, each of which has two sporozoites. The excystation of oocysts occurs on the exposure to trypsin (0.5%) and sodium taurocholate (1.5%) in phosphate-buffered saline, followed by mechanical disruption (Ortega et al., 1993). Based on the morphology and physicochemical properties of oocysts, electrorotation technique was developed for observing the changes in the oocysts (Dalton et al., 2001, 2004), but the technique is not handy due to procedural complexity and only can be used in research settings.

Oocyst sporulation and infectivity testing in animal model is an ideal method for evaluating the viability and infectivity of the oocysts (Giangaspero and Gasser, 2019). However, due to the unavailability of effective *in vitro* culture methods and *in vivo* animal models for *C. cayetanensis*, sporulation in 2.5% potassium dichromate is currently regarded as the only indicator for oocyst viability (Eberhard et al., 2000).

## Therapy

No vaccine is available for cyclosporiasis (Giangaspero and Gasser, 2019) but early detection and treatment can yield a favorable clinical outcome. Expectant treatment and chemotherapeutic treatment is crucial in human cyclosporiasis, particularly in immunodeficient individuals. Although case fatality due to cyclosporiasis is rare in humans, long lasting diarrhea sometimes results in dehydration or malnutrition, and occasionally may cause severe dehydration and death in infants (Behera et al., 2008; Bednarska et al., 2015).

Chemotherapy including treatment with 160 mg trimethoprim and 800 mg sulfamethoxazole (TMP-SMX, also known as co-trimoxazole) twice daily for 7 days can reportedly cure human cyclosporiasis (Hoge et al., 1995; Escobedo et al., 2009). TMP-SMX is considered as an effective drug, with many studies reporting low recurrence rates (Hoge et al., 1995; Madico et al., 1997; Goldberg and Bishara, 2012). It is also an effective chemotherapeutic treatment for *C. cayetanensis* infection in AIDS patients (Pape et al., 1994; Verdier et al., 2000) and those of them with biliary disease (Sifuentes-Osornio et al., 1995).

In some patients, TMP-SMX creates intolerance and allergy. In such cases, ciprofloxacin antibiotic with having less effectivity than TMP-SMX is a suitable treatment option for cyclosporiasis in human (Verdier et al., 2000). Nitazoxanide is another drug that can also be used in the cases of sulfonamide intolerance and ciprofloxacin resistance (Diaz et al., 2003; Cohen, 2005; Zimmer et al., 2007). Nitazoxanide has been used to treat mixed parasite infection with intestinal protozoa (including *C. cayetanensis*) and helminths (Diaz et al., 2003). The efficacy of nitazoxanide for cyclosporiasis was reported to be ranging from 71 to 87%. The tolerance level of the drug was found to be very high with having no serious adverse effects (Table 2). Conversely, norfloxacin, metronidazole, tinidazole, and quinacrine have proven ineffective in some studies of human cyclosporiasis (Escobedo et al., 2009; Almeria et al., 2019). In a more recent study in mice silver nanoparticles were effective against *Cyclospora* infection (Gaafar et al., 2019). This will draw attention to its potential for use as an alternative to the standard therapy in both immunocompetent and immunosuppressed hosts.

**Table 2:** Anti-*Cyclospora* oocyst drugs.

**Drugs**	**Dosages**	**Applicable population (scope)**	**References**
TMP-SMX	(160 mg trimethoprim, 800 mg sulphamethoxazole) twice daily for 7 days	AIDS patients and those of them with biliary disease; an effective treatment with a low recurrence rate	Hoge et al., 1995; Madico et al., 1997; Goldberg and Bishara, 2012
Ciprofloxacin	500 mg twice daily for 7 days	Patients with intolerance to sulfonamide drugs	Verdier et al., 2000
Nitazoxanide	100 mg (9.52 mg/kg bwt) twice daily for 3 days	Patients having sulfur intolerance or for whom treatment with sulfa or ciprofloxacin has failed	Cohen, 2005; Zimmer et al., 2007
Silver nanoparticles (NPs)	10 μg/mice i. p. once daily for 7 days	Only in experimental mice; effectiveness against *Cyclospora* infection	Gaafar et al., 2019
Magnesium oxide (MgO) nanoparticles (NPs)^*^	12.5 mg/ml for 3 days	Anti-*Cyclospora* effect on both unsporulated and sporulated oocysts in food and water disinfectant treatment	Hussein et al., 2018

*TMP-SMX, trimethoprim-sulfamethoxazole (also known as co-trimoxazole)*.

* *For disinfection of food and water potentially containing oocysts*.

## Prevention

*Cyclospora cayetanensis* is contracted via a fecal-oral transmission cycle, and direct person-to-person transmission seems unlikely. In developed nations, *C. cayetanensis* infections can be common in people who travel to endemic areas of underdeveloped and developing countries and consume the contaminated food, specially fresh produce imported from that regions (Almeria et al., 2019). *Cyclospora cayetanensis* is mainly transmitted via feces contaminated food, water, and soil (Almeria et al., 2019). Therefore, improvement of personal hygiene and sanitary conditions can be a suitable preventive approach for *C. cayetanensis* infections because it obviously cuts off the fecal-oral route of transmission of the parasite in the endemic areas. The practice of not consuming of raw fresh produce, especially those supplied from endemic areas can avert the problem of cyclosporiasis in humans. Regular boiling and filtering of water necessary for drinking, food preparation, and washing of fresh produce can also prevent the infection (Almeria et al., 2019).

While usual sanitizers and disinfectants can not destroy *C. cayetanensis* and coccidia in general, some exploratory methods for removing or inactivating *C. cayetanensis* oocysts in fresh fruits and raw vegetables have been investigated (El Zawawy et al., 2010; Butot et al., 2018; Hussein et al., 2018). In one study magnesium oxide nanoparticles had a significant anti-*Cyclospora* effect on both unsporulated and sporulated oocysts, prompting speculation that it may be useful as a preventive agent in food and water disinfection treatment (Hussein et al., 2018).

Care should be taken to keep the fresh produce out of contamination at the field and packaging unit, and also from the farm workers to efferently prevent the *C. cayetanensis* infection in endemic areas. The practice of toilet use, hand washing after toilet use and before meal and proper disposal and treatment of human excreta are also important for prevention of cyclosporiasis. Any worker bearing the gastrointestinal diseases should not handle the vegetables or other produce. Other coccidiosis control measures can also be applied for the prevention and control of *C. cayetanensis* infections.

## Conclusions

In conclusion, there are some advances in clinical features, diagnosis and therapeutic intervention of cyclosporiasis, which primarily diagnosed by the important clinical symptoms of watery diarrhea, abdominal cramps, and bloating. Conventional and laboratory diagnosis usually involves microscopy examination of wet smears, staining tests (typically the modified acid-fast stain), fluorescence microscopy, serological testing, or advanced molecular testing for oocyst DNA in the human stool. No vaccine is available for cyclosporiasis, but early detection and treatment can yield a favorable clinical outcome. Detection in a transmission vehicle such as fresh produce, water, or soil is helpful for case-linkage and source-tracking during cyclosporiasis outbreaks. The sensitivity of *Cyclospora* detection can be increased by the concentration of oocysts obtained from clinical or biological samples. Treatment with TMP-SMX has proven effective for cyclosporiasis. Ciprofloxacin, although less effective than TMP-SMX, can suitably be used in patients having sulfur drug intolerance. Nitazoxanide is an alternative drug can be used in the cases of sulfur intolerance and ciprofloxacin resistance. The water- and food-borne parasite *Cyclospora* has epidemiologically been investigated only in few underprivileged communities and developed nations. More epidemiological research on cyclosporiasis in humans should be conducted at various locations around the world, to achieve a better understanding of its characteristics in this regard. Attempts should also be made to establish *in vitro* or *in vivo* methods for cultivating *C. cayetanensis*. Rapid, convenient, precise, and economical detection methods for diagnosis, as well as effective tracing methods should be developed to monitor the transmission of *C. cayetanensis* infection.

## Author Contributions

LZ provided the ideas. JL and ZC wrote the draft manuscript. MQ participated in the modification of manuscript. All the authors had read the final manuscript.

### Conflict of Interest

The authors declare that the research was conducted in the absence of any commercial or financial relationships that could be construed as a potential conflict of interest.
